# 多灶性磨玻璃影肺腺癌的临床及分子特征

**DOI:** 10.3779/j.issn.1009-3419.2018.03.07

**Published:** 2018-03-20

**Authors:** 洋 宋, 乃新 梁, 单青 李

**Affiliations:** 1 1100730 北京，中国医学科学院北京协和医学院北京协和医院胸外科 Department of Toracic Surgery, Chinese Academy of Medical Sciences & Peking Union Medical Colledge Hospital, Beijing 100730, China; 2 100084 北京，清华大学医学院 School of Medicine, Tsinghua University, Beijing 100084, China

**Keywords:** 多原发肺癌, 遗传异质性, EGFR, 气腔播散转移, Multiple primary lung cancer, Genetic heterogeneity, EGFR, Spread through air spaces (STAS)

## Abstract

随着人们对肺癌早期筛查的重视，肺多发磨玻璃影（multiple ground glass opacities, GGOs）的检出率逐年增高，以GGOs为表现的多灶肺腺癌也逐渐成为临床研究热点。其更多见于女性、非吸烟者，且无论是自然病程还是手术后的患者均有极佳预后。独特的临床特征提示其很可能是相对独立的一种疾病形式。从分子遗传学路径对其的探究发现，同一个体内的多个病灶间极可能有截然不同的克隆性特征，因此遗传异质性（genetic heterogeneity）是以GGOs为表现多灶肺腺癌最显著的分子遗传学特征。此特征可辅助原发多灶肺腺癌与肺癌肺内转移的鉴别诊断，也提示了对病灶进行分子遗传学检测的治疗意义。部分呈现出遗传相似性的GGOs病灶为气腔播散转移（spread through air spaces, STAS）理论提供了新的证据。

## Abstract (en)

Due to emphasis on early screening for lung cancer, the detection rate of multiple ground glass opacities (GGOs) on computed tomography (CT) image increases in recent years, and research on multifocal adenocarcinomas presented by GGOs has been thriving. It is more common in women and non-smokers and has excellent prognosis both in patients with natural history and afer surgery. Tese clinical features suggest that it is likely to be a distinct disease entity. From the perspective of molecular genetics, lesions in the same individual are likely to have distinct clonal features. Terefore, genetic heterogeneity is the most prominent feature of multifocal pulmonary adenocarcinomas with GGOs. Te genetic heterogeneity is expected to assist the diagnosis of multifocal pulmonary adenocarcinoma and intrapulmonary metastasis, and also suggests that genetic testing of the GGO lesions is of great therapeutic signifcance. Some GGO lesions may harvest the similar clonal feature, which provide new evidence for the theory of spread through air spaces (STAS).

肺部磨玻璃影(ground-glass opacity, GGO)，或称磨玻璃结节(ground-glass nodule, GGN)，是一个影像学概念，指在胸部CT中表现为密度较正常肺组织轻度增高的，局灶性、云雾状淡薄影，因外观似磨砂玻璃，得名"磨玻璃影"^[1]^。根据GGO内部是否含有实性成分，GGO可进一步分为纯磨玻璃结节(pure GGO, pGGO)，和部分实性结节(part-solid lesion)。pGGO及部分实性GGO总称为亚实性(subsolid)结节。GGO可能是肺内恶性肿瘤，但一些良性病变，包括炎症、出血、纤维化，也可在计算机断层扫描(computed tomography, CT)上表现为GGO^[2]^。

本文将主要讨论以肺部多发磨玻璃影(multipleground glass opacities, GGOs)为表现的多灶肺腺癌(multifocal pulmonary adenocarcinomas)。因人们对肺癌早期筛查的重视及肺部CT尤其是低剂量螺旋CT(low-dose CT, LDCT)的日渐普及，此类肺腺癌的检出率逐渐升高，同时也呈现出其独特的临床及分子遗传学特征^[3]^。

## 1 以GGOs为表现的多灶肺腺癌的临床特征

### 1.1 流行病学特点

与传统非小细胞肺癌(non-small cell lung cancer, NSCLC)对比，无论在亚洲还是北美的临床患者中，表现为GGOs的多灶肺腺癌都更多见于女性(60%-80%)。除性别差异，此类肺腺癌患者中非吸烟者的比例虽在多个报道中有所差异(30%-80%)，但总体高于肺癌全体中的吸烟者比例^[4-7]^。

### 1.2 病理学特征

表现为GGOs的肺内恶性肿瘤有相对稳定特异的病理学基础：GGO内仍可见到包括血管、气道在内的正常的肺实质组织，是因为此类肺内肿瘤在肿瘤形成过程中的生物学行为是上皮细胞沿肺泡壁生长(lepidic growth)，不浸润或突破肺泡壁结构，因此在部分文献中，也将表现为GGO的肺内肿瘤称为GG/L^[8]^。一般认为，具有此种病理生长方式的肿瘤多属早期肺腺癌，主要包括微浸润腺癌(minimally invasive adenocarcinoma, MIA)、原位腺癌(adenocarcinoma *in situ*, AIS)、甚至非典型腺瘤样增生(atypical adenomatous hyperplasia, AAH)^[9]^。GGO内出现实性成分，往往提示更有侵袭性的病理类型^[10]^。

### 1.3 预后特点

表现为GGOs的多灶肺腺癌的临床转归也体现了其较为独特的生物学行为特征。从自然病程的角度来说：在3个分别纳入了28个、23个、23个患者，共计40个、89个、196个GGO病灶，中位随访24个月、40个月、49个月的临床研究中，60%-95%的pGGO病灶在无临床干预的条件下保持无变化，仅有若干个病灶减小或消失，若干个病灶增大或变成了需要手术切除的部分实性病灶(part-solid GGO)^[11-13]^。此结论与一篇综合讨论亚实性(subsolid)结节自然史(natural history)的综述报道的数据高度吻合：大多数的亚实性GGOs可保持临床稳定，约20%的病灶可实现体积减小甚至消失，20%的病灶可能增大或获得更多的实行成分(中位随访期：9个月-50个月)^[14]^。

手术后的肿瘤性GGOs也有极佳预后。2017年，Chen^[15]^进行了一项包括96例多原发肺癌的患者的研究，其中包括表现为GGOs的24例患者(定义为实性成分在肿瘤病灶中的占比≤50%)，共计51个病灶。所有GGO病灶病理类型均为肺腺癌，且在70个月的随访期内，该组表现为GGOs的肺腺癌患者手术后无复发生存率为100%。此类患者与单发肺内肿瘤患者的术后预后没有显著差异^[16]^。据国际肺癌研究协会(International Association for the Study of Lung Cancer, IASLC)报告，手术后GGOs的5年生存率可达90%，且与非小细胞肺癌的总体数据相比，有显著更低的淋巴结或远处转移倾向^[8]^。

综上，表现为GGOs的多灶肺腺癌的预后特点提示了其偏惰性的生物学行为特征。因此，2016年IASLC建议在第八版美国癌症分期联合委员会(American Joint Commitee on Cancer, AJCC)肿瘤-淋巴结-转移(tumor-node-metastasis, TNM)分期中对其应用特殊的TNM分期^[8]^：据T分期最高的单个病灶制定肺内所有结节的T分期，在其后的括号内标注病灶个数，并用一个N分期和一个M分期共同表述所有结节的转移情况，如"T1a(4)N0M0"。

基于表现为GGOs的多灶肺腺癌独特的病理及临床特征，此类肺腺癌应被视为相对独立的一种疾病形式逐渐成为共识，对于此类肺腺癌分子遗传学特征的探究也逐渐兴起。

## 2 分子遗传学特征及其临床意义

### 2.1 表现为GGOs的多灶肺腺癌的遗传异质性

对表现为GGOs的多灶肺腺癌的分子遗传学特征探究主要围绕着多个肺癌的"标记基因(biomarker)"进行，即肺癌发生过程中早期发生、高频发生突变的基因，此类基因对于探究肺癌病灶的成因及克隆性关系有重要意义。主要包括*EGFR*、*KRS*、*ALK*等(表 1)。

**1:** 表现为GGOs的多灶肺腺癌的遗传异质性特征 Genetic heterogeneity of multifocal pulmonary adenocarcinomas presented by GGOs

First author	Country, year of publication	Number of patients, GGO leisions	Gene testd	Lesions with *EGFR* mutation(%)	Genetic heterogeneity (Discordance rate)
Liu^[22]^	China, 2016	78, 159	*EGFR*	49	Discordance rate of *EGFR* mutation in 38 paired lesions: 92%
Takamochi^[23]^	Japan, 2012	36, 82	*EGFR*, *KRAS*	44	Discordance rate of *EGFR* mutation in 20 paired lesions: 91%
					Discordance rate of *KRAS* mutation in 15 paired lesions: 93%
Chung^[24]^	Korea, 2009	18, 44	*EGFR*, *KRAS*	41	Discordance rate of *EGFR* mutation: 72%
Wu^[25]^	China, 2015	35, 72^*^	*EGFR*, *KRAS*, *HER2*, *BRAF*, *PIK3CA*, *ALK*, *ROS1*, *RET*	45.8	Discordance rate of driver gene mutation: 80%
Chen^[15]^	China, 2017	23, 48^	*EGFR*, *KRAS*, *TP53*, *BRAF*, *PIK3CA*, *ALK*, *ROS1*, *RET*	47.9	Discordance rate of driver gene mutation: 100%
Multifocal adenocarcinomas with GGOs is a hot research topic in recent years, and nearly 50% of the GGO lesions harvest an *EGFR* mutation in the Asian population. Moreover, under the same genetic and environmental background, GGO lesions with genetic heterogenetiy may occur in the same individual. ^*^12 solid nodules were included in Wu's study; ^One of the recruited patients presented with wild type in all the driver genes and was not included in the discordance rate calculation.					

*EGFR*是重要的肺癌标记基因，其酪氨酸激酶结构域的突变与细胞的恶性增殖有直接关系，也因此是靶向药物[酪氨酸激酶抑制剂(tyrosine kinase inhibitors, TKI)]的重要靶点^[17, 18]^。同时，EGFR在亚洲肺腺癌患者中较美^[19]^、德国^[20]^人群有显著较高的突变率^[21]^，因此也是亚洲GGOs患者队列中供病灶遗传异质性鉴别的关键位点。2016年Liu^[22]^进行了一项纳入78例GGOs患者的临床研究，每例患者均有至少2处病灶进行了手术切除，证实为多原发肺腺癌。此临床队列中近50%的患者至少有一处病灶有*EGFR*突变，仅有8%的患者的两处癌灶有相同的EGFR突变。此项研究样本量大，与其他两项在日^[23]^、韩^[24]^进行的研究报道了一致结论，提示GGOs的病灶间有着巨大的遗传异质性(genetic heterogeneity)。

除此项研究，还有若干项其他研究从分子遗传学的不同侧面对GGOs病灶间遗传异质性进行研究。Wu^[25]^和Chen^[15]^对肺癌的8个经典驱动基因进行研究(*EGFR*、*KRAS*、*BRAF*、*PIK3CA*、*ALK*、*ROS1*、*RET*、*HER2*/*TP53*)，发现表现为GGOs的多灶肺腺癌病灶间驱动突变的异质性可达80%-100%^[15, 25]^。

综上，在遗传背景及外界环境完全相同的情况下，同一人体内可出现克隆性特征(基因图谱)截然不同的肿瘤灶。遗传异质性是表现为GGOs的多灶肺腺癌最显著的分子遗传学特征。

### 2.2 遗传异质性的临床意义

#### 2.2.1 诊断

目前对于GGOs的诊断仍主要基于影像及病理学判断，也因此有较大的主观性^[26]^。然而如前文所述，标记基因的突变在肺癌发生过程中是早期发生、高频发生，且随机发生的突变，因此已有多个研究报道，GGOs癌灶间标记基因克隆性的不一致可与临床病理学诊断互补，辅助原发多灶肺腺癌与肺癌的肺内转移的鉴别诊断，也将使上文述及的对于表现为GGOs的多灶肺腺癌的TNM分期更为精确。在众多讨论多灶肺腺癌的临床指南中，均强调了GGOs病灶分子遗传学特征的诊断价值^[3]^，有待于将来更多的研究将其临床应用定量化、规范化。

#### 2.2.2 治疗

对于肺内病灶进行分子标记物检测已是肺癌临床管理中的成熟操作。但表现为GGOs的多灶肺腺癌作为相对特殊的一种疾病形式，在其基因诊断策略上仍有若干尚待回答的问题。

上文提及的Liu^[27]^的研究中，仅19%的患者的每个癌灶中均有*EGFR*的突变(尽管具体突变位点可能并不相同)。也即是说，此临床队列中，仅有19 %的患者在应用针对*EGFR*突变的TKI药物后，所有癌灶的恶性增殖均能得到控制。但同时，即便在亚裔GGOs患者中有近乎50%的癌灶有*EGFR*突变^[15, 25, 27]^，但由于临床工作中对每个病灶都进行检测并不实际，无法确保有*EGFR*突变的病灶被检测到。虽然此类GGOs的患者仅有具有*EGFR*突变的病灶可以从TKI治疗中部分获益，但可能因为没有检测到有突变的病灶，而没有应用TKI药物的指征。因此，是否需要对其多个病灶的每个病灶都分别进行基因检测，才能找到可指导靶向治疗的所有靶突变、彻底除外靶向治疗的有效性或评价靶向治疗的有效性，仍是一个尚待回答的问题。

此外，既往临床工作中曾有*KRAS*与*EGFR*的突变相对互斥(mutually exclusive)的共识^[21]^，因此在测得*KRAS*突变后不再检测*EGFR*突变。但病灶间突变基因的异质性使临床工作者开始对这个既往理念产生疑问。事实上，已经有在同一个体同时发现*EGFR*与*KRAS*突变的直接证据^[28]^，这再次强调了需要对每个GGO病灶进行基因检测可能的必要性。

#### 2.2.3 具有遗传相似性GGOs的部分患者

无论是在2013年Fleishner学会^[2]^，还是2016年IASLC给出的关于GGOs的临床指南中^[8]^，均将以GGOs为表现得多灶肺腺癌描述为肺癌发生发展中的早期事件，也因此多发GGOs患者常常除外肺内转移，倾向于被诊断为多原发肺癌。Li^[29]^对两个多灶肺腺癌的GGOs患者进行外显子测序研究，首先报道GGO病灶与TCGA中肺腺癌的数据相比，确有较低的突变负荷，支持其是早期肺癌的共识；但该课题组同时报道了这两个患者的成对GGO病灶之间存在高度相似的体细胞突变方式，且均无淋巴转移或血行转移证据。此案例为肺癌气腔播散转移(spread through air spaces, STAS)概念提供了新的支持^[30, 31]^，也可能对部分肿瘤性GGOs患者较差的预后提出解释。从治疗的角度，对这部分表现为GGOs的多灶肺腺癌患者，楔形切除可能无法达标，还需要考虑更广泛的肺段甚至是肺叶切除，甚至是术后的全身治疗。

## 3 结语

表现为GGOs的多灶肺腺癌有独特的流行病学、病理学、预后特征，提示了其是一种相对独立的一种疾病形式。其分子遗传学层面的突出特征是多病灶间的遗传异质性，可辅助判断病灶的克隆性来源；部分呈现出遗传相似性的GGOs病灶则为气腔播散转移提供了新的证据。对病灶进行分子遗传学检测的临床意义将随着基础医学探索的深入，逐渐得到揭示。
